# Precision-Cut Tumor Slices (PCTS) as an Ex Vivo Model in Immunotherapy Research

**DOI:** 10.3390/antib11020026

**Published:** 2022-04-06

**Authors:** Paraskevi Dimou, Sumita Trivedi, Maria Liousia, Reena R. D’Souza, Astero Klampatsa

**Affiliations:** 1 Division of Cancer Therapeutics, The Institute of Cancer Research, London SM2 5NG, UK; vivian.dimou@icr.ac.uk (P.D.); reena.dsouza@icr.ac.uk (R.R.D.); 2 Division of Hematology and Medical Oncology, Department of Medicine, University of Pennsylvania, Perelman School of Medicine, Philadelphia, PA 19104, USA; sumita.trivedi@pennmedicine.upenn.edu; 3 Pulmonary, Allergy and Critical Care Division, Department of Medicine, University of Pennsylvania, Perelman School of Medicine, Philadelphia, PA 19104, USA; mliousia@pennmedicine.upenn.edu

**Keywords:** precision-cut tumor slices, PCTS, organotypic tumor slices, immunotherapy, cytotoxic T cells, CAR-T cells, ex vivo models, solid tumors

## Abstract

Precision-cut tumor slices (PCTS) have recently emerged as important ex vivo human tumor models, offering the opportunity to study individual patient responses to targeted immunotherapies, including CAR-T cell therapies. In this review, an outline of different human tumor models available in laboratory settings is provided, with a focus on the unique characteristics of PCTS. Standard PCTS generation and maintenance procedures are outlined, followed by an in-depth overview of PCTS utilization in preclinical research aiming to better understand the unique functional characteristics of cytotoxic T cells within human tumors. Furthermore, recent studies using PCTS as an ex vivo model for predicting patient responses to immunotherapies and other targeted therapies against solid tumors are thoroughly presented. Finally, the advantages and limitations of the PCTS models are discussed. PCTS are expected to gain momentum and be fully utilized as a significant tool towards better patient stratification and personalized medicine.

## 1. Introduction

The recent advances in immunotherapies, such as immune checkpoint modulators and adoptive T-cell transfer, open new opportunities for the treatment of cancer. With this broad spectrum of new therapeutic agents available, however, the need for robust, predictive preclinical models to minimize translational failures in immuno-oncology is increasing.

Several preclinical mouse models have been developed throughout the years to model human cancer and predict patient responses. These mouse models carry important limitations, as they are unable to fully recapitulate human tumor genetics and histology as well as the human tumor microenvironment (TME) [1,2]. For this reason, more advanced human models of cancer disease are urgently necessary.

The ex vivo generation and culture of live precision-cut tumor slices (PCTS) deriving from various human solid tumor sites has lately emerged as a potential tool for successful personalized drug screening [3,4]. PCTS, or “organotypic tumor slices”, are found at the crossroads of in vitro and in vivo tumor models, combining advantages both regarding time-efficiency and accurate 3D tumor architecture and TME recapitulation.

This review delves into the important role of PCTS slices in modern cancer research with a focus in immunotherapy, as well as in the progress of patient stratification and personalized medicine.

## 2. Established In Vitro, In Vivo and Ex Vivo Experimental Tumor Models

### 2.1. Three-Dimensional Tumor Cell Cultures

Within laboratory settings, several standardized human tumor models have been widely used throughout the years to study the biology of cancers [5]. The most used model is that of the in vitro culture of 2D monolayers of cancer cells that can be either primary tumor cells or, most often, established, immortalized tumor cell lines. The latter are derived from expanded primary tumor cell clones and allow for the generation and freezing of several passages, offering scientists a readily available, easy to manipulate tool for day-to-day in vitro experimental assays. Although simple to use, easily expandable and able to be maintained long-term in culture, the 2D monolayers of tumor cell lines lack the 3D structure of real tumors. The formation of intricate 3D tumor architecture in vivo allows for cell-to-cell interaction and signaling, resulting in advanced resistance to treatment [3]. Thus, the effect of different anti-tumor agents can often be significantly weaker in 3D tumors compared to 2D monolayers [6,7].

### 2.2. Three-Dimensional Human Tumor Models

The culture of tumor cell lines in specialized collagen or other gel-like matrices allows for tumor cells to obtain a more 3D, spherical-like shape [3,8,9]. These 3D tumor “spheroids” are relatively easy to maintain in culture and can be more accurately used in drug treatment assays [7]. Nonetheless, both 2D and 3D cell line-based tumor models lack the in vivo tumor heterogeneity, where different cells within the same tumor site can have quite distinct and diverse genetic, epigenetic, phenotypic and tumor antigen expression profiles. To this end, the recent emergence of patient-derived organoids (PDOs) aims to predict patient response to chemotherapeutic, immunotherapeutic, and other targeted regimens across different cancer types (breast, lung, colorectal, gastrointestinal, and other cancers) [10,11,12,13]. PDOs are generated from patient solid tumor tissue, which is enzymatically digested to obtain tumor cells that are then cultured ex vivo in specialized basement membrane matrices allowing them to form 3D, tumor-resembling, organ-specific structures [14]. PDOs are a superior model to 2D tumor cell line monolayers and 3D tumor spheroids derived from established tumor cell lines, as they more accurately represent the high tumor genetic heterogeneity, the differences in mutational landscape and metastatic potential of tumor cells across different cancer patients [14]. However, none of them retains the highly complex and heterogeneous TME and stroma, as only isolated tumor cells are used for their development. For this reason, patient-derived xenograft (PDX) murine models have been developed.

### 2.3. PDX Murine Models

PDX models are developed via subcutaneous or organ-specific implantation of fresh patient tumor tissue or cells in immune-deficient mice. The engraftment of primary tumor cells or fragments results in the development of nodules that retain the phenotypic characteristics of the original tumor^.^ They are used in various applications, including the preclinical testing of clinically available therapies to determine optimal combinations for individual patients, predictive biomarker discovery, drug screening and the elucidation of mechanisms leading to drug resistance [15]. Despite the big advantages of PDX models, their drawbacks render them difficult to use on a regular basis in preclinical and clinical settings. PDX models are quite costly, highly laborious and time consuming, necessitating several weeks or even months until ready for use in experimental assays [3,15]. Furthermore, initial patient tumor engraftment is not always guaranteed to be successful [3,15]. Most importantly, the lack of an immune system in PDX models prevents the study of the immune TME and the screening of novel immunotherapeutic agents [3].

## 3. Precision-Cut Tumor Slices (PCTS) as an Ex Vivo Platform in Immunotherapy Research

**Figure 1:**
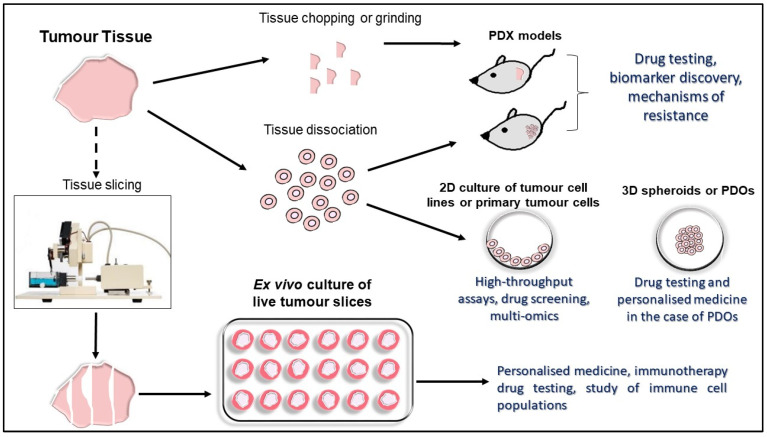
Overview and main applications of preclinical tumor models.

PCTS are an ex vivo platform able to recapitulate the high in vivo intratumoral complexity more accurately than other existing models [4,16]. PCTS are generated via the cutting of thin slices of fresh tumors without prior processing, thus preserving the tumor architecture, stroma and the diverse cell populations of the TME. Indeed, a study using pancreatic ductal adenocarcinoma (PDA) tumor slices generated following patient surgery showed that components of the TME, including T cells and macrophages, were able to survive in PDA slices cultured for over a week, allowing for studies of the PDA immune landscape [17]. Early drug screening findings on PCTS have shown results similar to those of PDOs, with the additional advantages that PCTS generation requires minimal manipulation, allows for faster results and offers the chance to study patient responses to various novel immunotherapies [18]. Figure 1 summarizes the preclinical human tumor models currently being used within laboratory settings.

### 3.1. Vibratome Technology for PCTS Generation

PCTS are generated using vibrating microtomes, known as “vibratomes”. Microtomes, used extensively in diagnostic histopathology, are relatively simple instruments comprising of a blade and a blade holder, an object clamp used to hold and secure objects, an advancement mechanism, and a mechanism for section thickness adjustment that allows for precise control [19]. The way in which most microtomes work is by moving the sample over the blade, leading to the advance mechanism automatically moving forward in a way that all allows for precision slicing at a desired thickness [19]. There are several types of microtomes, each of them specialized in optimally slicing different types of specimens (summarized on Table 1) [19].

Vibratomes incorporate vibration to the microtome blade, resulting in less pressure and stress to the tissue sample than a stationary blade, and are therefore ideal for fresh tissue slicing [3]. In the early years of PCTS generation, the Krumdieck tissue slicer, a type of rotary microtome, was used [20]. Although it is still considered a better choice for slicing certain tumors, such as glioblastomas [21], vibratomes have been shown to cut tissue slices more precisely and reproducibly [22]. The most important vibratomes currently in market are the Leica Vibratome Series (Leica Biosystems) and the Compresstome Series (Precisionary Instruments Inc., Greenville, NC, USA). The Compresstome has been developed to cut fresh and fixed tissue slices with very mild compression of the tissue, helping to avoid sliced tissue damage and shearing and prevent uneven slicing or vibration-related artefacts [23,24]. The Vibratome and Compresstome were both shown to produce high quality PCTS [23], with the Compresstome having important advantages such as easier slicing and with a higher speed than the Vibratome [23].

### 3.2. Generation and Culture of PCTS

The generation of PCTS involves the acquisition of adequate volume of fresh tissue, usually provided as a surplus to diagnostic procedures. The production of PCTS occurs within a few hours post-surgery to promote tissue survival and retain high levels of tissue viability [3,18].

**Figure 2:**
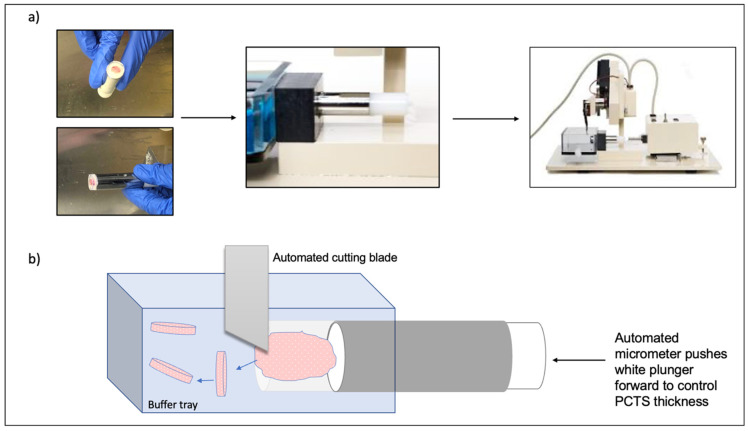
PCTS generation using the Compresstome. (**a**) Tumor samples are embedded in agarose inside specialized tube, on top of a plunger. The tube is then cooled and, once the agarose is solidified, the tube is secured on the Compresstome with the tissue side of the tube being within a buffer tray. (**b**) An automated cutting blade produces serial PCTS, which are free-floating in the buffer tray. To control PCTS thickness, an automated micrometer pushes the specimen tubes white plunger forward after each blade cut.

Fresh tissue samples are placed in ice-cold tissue storage buffers (such as PBS, HBSS, media, MACS tissue storage buffer, or Belzer UW Cold Storage Solution) immediately following surgical excision. Once in the laboratory and using a biopsy punch, several cores can be obtained from the tissue sample [18,25]. Alternatively, the tissue can be manually cut into shape with a sterile scalpel [26]. Subsequently, tissue processing to allow PCTS generation generally involves tissue embedding in an embedding mold, the addition of warm, low-melting agarose to cover the tissue and then cooling with ice-cold media. Once the agarose is solidified and the mounted tissue sample is secured within it, it is taken out of the mold, secured in place on the vibratome and PCTS can be generated (Figure 2) [18,25].

Tissue slicing speed (mm/s) and vibration amplitude (mm) are selected according to tissue type, density, consistency and integrity [18]. Once PCTS are generated, they are removed from the tray using sterile forceps and are placed on desired culture plates. Several detailed protocols exist, describing the process of PCTS generation and subsequent culture [17,18,27,28,29,30,31,32].

PCTS can usually retain their viability as well as the same histological, architectural, and phenotypical characteristics as the original tumor for an average of 6–7 days, depending on tumor type [3,4,18,31,33,34]. Thus, the ex vivo testing of therapeutic agents on PCTS can occur generally within this timeframe. Their thickness can vary from 30 μm to 1000 μm and is selected based on tumor type and ease of slicing, as well as the effective diffusion and penetration of drugs and nutrients [18]. Simple culture media, such as DMEM or RPMI-1640 supplemented with fetal bovine serum (FBS) and antibiotics (penicillin/streptomycin), suffice for maintaining viable PCTS, with organ-specific growth factors added if necessary. PCTS have been cultured in tissue culture plates or on 0.4 μm pore Teflon membrane inserts, which have shown to be better in overall PCTS preservation and survival [35,36]. A study of breast PCTS cultured under constant rotation has shown increased viability compared to static culture conditions [34].

## 4. What Is the Value of PCTS in Immunotherapy Research?

### 4.1. PCTS as a Model for Studying Localisation and Function of the Immune TME

PCTS offer the opportunity to study the localization and function of the several immune cell types within the TME, and how the latter behave following treatment. With CD8+ cytotoxic and CD4+ helper T cells playing a central role in anti-tumor immunity, several PCTS-based studies have focused on understanding the localization and function of tumor infiltrating lymphocytes (TILs) in solid tumors, including lung, breast and melanoma [27,37,38,39,40,41,42,43,44,45,46,47].

CD103+ CD45RO+ CD8+ tissue resident memory T cells (Trms) have recently emerged as a highly cytotoxic CD8+ T-cell subset localized within mucosal tissues and playing a key role in anti-tumor immunity in several solid tumors [41,42,43,44,45,46]. In 2016, a study utilizing human lung cancer PCTS, autologous tumor antigen-specific TILs and peripheral blood T cells (PBTs) showed that CD103-expressing CD8+ T cells had the ability to infiltrate epithelial tumor islets, in contrast to CD103-negative CD8+ cells, which preferentially accumulated in the tumor stroma [37]. The addition of TGFβ in the autologous cultures increased CD8+ CD103 expression and enhanced tumor infiltration [37]. In a more recent study, 100–200 μm-thick PCTS from a breast cancer PDX model used in imaging assays revealed that CD49a integrin expression increased the motility of CD8+ TILs that were near tumor cells, potentially distracting them from tumor antigen recognition [40]. In addition, there was evidence of CD49b expression resulting in CD8+ TIL relocation to areas more distant to the tumor cells [40]. Adequate T-cell tumor infiltration is necessary for successful tumor elimination. In a study by Salmon et al., 400 μm thick PCTS derived from NSCLC patients following surgical resections were immunostained for the detection of T cells (CD3 marker), tumor cells (EpCAM marker for epithelial cell adhesion) and surrounding stroma (CD90 and fibronectin markers for fibroblasts, endothelial cells and the extracellular matrix) [38]. Using real-time fluorescence microscopy, it was demonstrated that TILs preferentially accumulate in the stroma, rather than the tumor-cell-rich regions [38]. To assess the potential of T-cell migration within the PCTS, in vitro activated and fluorescently labelled PBTs as well as autologous TILs were added to the PCTS. As the Trms, the PBTs and TILs preferentially accumulated in the stroma area [38]. Furthermore, using 2-photon imaging and second-harmonic generation (SHG) microscopy combined with immunostaining, it was demonstrated that T cells preferentially accumulated in collagen-sparse regions [38]. Thus, when the PCTS were treated with collagenase, added PBTs were more motile and more efficiently infiltrated the stromal regions adjacent to the tumor-cell-rich areas to reach tumor islets, indicating the therapeutic potential of targeting collagen in NSCLC [38].

In a similar study by the same group, the motility and migration patterns of CD8+ TILs within lung and ovarian cancer PCTS were explored [47]. Immunostaining for TILs (CD8+) and tumor cells (EpCAM+) and time-lapse confocal microscopy was combined with SHG imaging for the tumor stroma to reveal that, as lung PCTS, TILs in ovarian PCTS preferentially accumulated within stroma regions and were less abundant in tumor islets [47]. Nonetheless, TILs close to tumor regions moved and migrated faster than those located in the stroma [47]. Collagen fibers were found to be involved in the downregulation of T-cell motility and were inversely correlated with the presence of CD8+ T cells within the stroma [47]. This work indicated that immune checkpoint inhibition and restoration of T-cell function might not be enough as a therapeutic approach, as the ability of CD8+ T cells to reach closer to the tumor islets and efficiently infiltrate them is an equally important parameters for them to successfully exert their anti-tumor functions.

Data from such studies can be helpful in identifying factors that block spatial distribution of TILs within tumor cell-rich areas, as well as factors with a role in TIL anergy, ultimately contributing to the discovery of biomarkers of response to immunotherapies.

### 4.2. PCTS as a Platform to Assess Chimeric Antigen Receptor (CAR) T Cell Infiltration and Activation

PCTS use in CAR-T cell research is still in its infancy. A 2021 study used EGFR-targeting CAR-T cells in co-cultures with either PCTS derived either from a BxPC3 EGFR-expressing pancreatic cancer PDX model or from PCTS derived from NSCLC and ccRC [39]. Confocal microscopy and dynamic imaging techniques were used to monitor CAR-T-cell migration and infiltration in the PCTS, whereas fluorescent calcium assay assessed CAR-T cell activation [39]. The results demonstrated CAR-T cell-specific activation that was dependent on the presence of EGFR-expressing tumor cells at the periphery of the tumor islets, which are initially the only ones permissive to CAR-T cell binding [39]. In response to EGFR CAR-T cell activation and IFN-γ secretion, the tumor cells upregulated the expression of the intercellular adhesion molecule-1 (ICAM-1), which in turn enabled the progressive entry of EGFR CAR-T cells from the periphery to the center of tumor islets [39]. The importance of ICAM-1 expression by the tumor cells was further highlighted by the fact that EGFR-expressing tumor cells at the center of the tumor islets that weakly expressed ICAM-1 were not able to promote EGFR CAR-T cell activation “arrest” within the tumor islets [39]. Additionally, researchers were able to show that, despite the weak expression of ICAM-1 by tumor cells, the presence of adequately high numbers of CAR-T cells can upregulate the expression of ICAM-1 via increased IFN-γ production, rendering tumors more permissive to effector T-cell infiltration [39]. Thus, this study highlighted the importance and critical role of ICAM-1 in predicting the effectiveness of CAR-T cell therapies as well as the key role of IFN-γ in turning immunologically “cold” tumors “hot” [39].

### 4.3. Use of PCTS in Predicting Patient Response to Immunotherapy

The potential of PCTS in predicting patient response has been under evaluation for several years. Most studies to date have used PCTS to assess patient responses to chemotherapy and targeted therapy [34,48,49]. Using PCTS from NSCLC patients, the effect of the nanoparticle-mediated delivery of an antisense 2′-O-methyl-RNA oligonucleotide on the ability to inhibit telomerase activity has been assessed [49]. Naipal et al. used breast cancer PCTS to test differential responses to the clinically approved chemotherapeutic FAC regimen (combination of 5-FU, Adriamycin [Doxorubicin] and Cyclophosphamide) and accordingly distinguish patients to FAC sensitive and FAC resistant [34]. The researchers’ findings were in accordance with one clinically proven therapy resistant tumor, suggesting that PCTS could be a reliable preclinical model for swiftly identifying breast cancer patients who might not benefit from FAC therapy [34]. In another study published in 2016, ex vivo generated PCTS derived from murine PDX models of PDA were used for the prediction of patient response to clinically relevant anti-PDA chemotherapeutic agents, including gemcitabine, irinotecan, MEK inhibitor AZD6244 and AKT inhibitor MK2206 [48]. The retrospective analysis of patient clinical follow-up data revealed that patients whose PCTS were sensitive to gemcitabine and irinotecan also exhibited satisfactory responses to these chemotherapeutic agents in clinic [48]. In a 2018 study, researchers developed three different prostate cancer (PC) PDX models characterized by different androgen receptor (AR) expression and breast cancer associated two (BRCA2) status (wild type versus mutated) [50]. PCTS deriving from these models were shown to be an effective platform for the prediction of patient response to the targeted therapies enzalutamide and olaparib [50].

More recently, PCTS from nine hepatic metastatic colorectal cancer patients were treated with clinically relevant oxaliplatin (chemotherapy), cetuximab (EGF inhibitor) and pembrolizumab (PD-1 inhibitor) [51]. It was demonstrated that eight out of nine PCTS samples were susceptible to oxaliplatin in a dose-dependent manner, a fact that resonates with the use of chemotherapies as a first-line treatment in clinic. In addition, only two patient responses to cetuximab and pembrolizumab were noted, respectively, indicating the potential of PCTS as preclinical models to distinguish responders and non-responders to targeted immunotherapies [51].

Seo et al. generated PCTS from surgical resections of patient PDA tumors [52] and demonstrated that larger numbers of TILs tended to accumulate in stromal regions containing fibroblasts, but devoid of carcinoma cells [52]. Smaller numbers of TILs were present in highly immunosuppressive juxtatumoral regions that were rich with carcinoma cells, macrophages, and regulatory T cells (Tregs) [52]. Given the lack of satisfactory PDA patient responses to checkpoint inhibitor therapies, the combined treatment with anti-PD-1 antibody and anti-CXCR4 small molecule inhibitor was tested on the PDA PCTS [52]. CXCR4 is highly expressed in PDA and is also expressed by T cells, leading to their immobilization within tumor sites via CXCR4 binding to the stromal derived factor-1 (SDF-1) or else known as CXCL12. Thus, the combined blockade of CXCR4 and PD-1 led to highest levels of CD8+ T-cell mobilization and migration towards the carcinoma cell-rich juxtatumoral stroma regions, as observed via live microcopy [52]. Increased tumor cell death was observed following combination treatment and, in addition, the enzymatic dissociation of PCTS and subsequent flow cytometric analysis showed the increased presence of CD45+, CD8+ and CD4+ T cells after treatment combination [52]. Therefore, the study highlighted the effectiveness of a clinically relevant combination treatment in PDA via the use of patient derived PCTS as a preclinical model [52].

In a 2020 study, researchers treated PCTS derived from clear cell renal carcinoma (ccRC) patient surgical resections with either emsirolimus (mTOR inhibitor blocking cancer cell proliferation), pazopanib (angiogenesis blockade), or sunitinib (blockade of angiogenesis and tumor cell proliferation) to explore differences in drug sensitivity of different patients’ PCTS [26]. Indeed, PCTS with distinct drug response profiles were identified, indicating that PCTS could be an efficient preclinical model for guiding optimal therapeutic choices in ccRC [26]. The PCTS were additionally used to assess TIL presence, as the latter is an important predictor of ccRC patient prognosis and response to immunotherapies [26].

A recent study by Horowitz et al. aimed at developing a more automated, digitally manufactured microfluidic platform, allowing for multiplexed drug testing using intact glioblastoma (GBM) PCTS derived from patients or PDX models [53]. This microfluidic platform consists of a 40-channel device manufactured from PMMA (poly (methyl methacrylate)) that allows for multiplex testing of at least 20 different drug conditions using the same tissue slice [53]. This platform allowed tumor slice survival and preserved stromal and TME for at least 4–5 days [53]. This promising application of the microfluidic platform could pave the way for the development of more high-throughput PCTS platforms in the future, allowing for multiple drug testing in a cost-effective and time-efficient manner.

## 5. PCTS: Advantages and Limitations

PCTS are highlighted as the ex vivo model that fully retains the real tumor 3D landscape, along with its TME, including stroma and immune cells. They are relatively easy to produce and, within their viability timeframe of up to a week, can offer a platform to test tumor responses of immunotherapies, such as checkpoint inhibitor antibodies and CAR-T cells. As CAR-T cells are a “living drug”, ex vivo PCTS models can be used to study CAR-T cell kinetics, spatial distribution, and migration patterns, additional to their function. Thus, within the next decade, more preclinical PCTS-based research studies on CAR-T cells are expected to emerge.

PCTS present with several limitations, such as the inability to be cultured long-term and to be biobanked due to current limitations in the PCTS freezing processes (although this may change soon). Another important limitation is that PCTS are low throughput systems. Tissue dissociation leading to tumor and immune cell isolation allows for high-throughput assays, such as transcriptomics, multi-omics, single-cell profiling, multi-color flow cytometry and multi-panel drug testing. In contrast, PCTS allow for limited drug testing each time (and thus, careful selection of the therapeutic agents to be tested is necessary), and for imaging and immunohistochemistry techniques that usually permit the testing of only a small number of fluorescent markers. The development of commercial microfluidic systems in the future could lead to platforms capable of supporting multiplexed drug testing in live tumor slices.

Although PCTS cannot be used directly for multi-omics analyses, they can still be dissociated or PCTS lysates can be generated and can subsequently be used for transcriptomic, proteomic and metabolomic assays [54,55,56]. A recent study using human lung PCTS followed a novel approach for PCTS RNA isolation, which involved agarose separation (PCTS are embedded in low-melting agarose, which can affect RNA quality) [56]. This approach led to RNA isolation of high yield and quality, which was successfully used in qRT-PCR and RNA sequencing studies [54,56]. Bulk RNA sequencing data from this approach were successfully used in deconvolution computational pipelines that led to the detection of various cell populations (including immune cell populations) within the human lung PCTS [54,56]. Furthermore, another recent work using human lung PCTS provided evidence for a successful integrated approach for the measurement of pro-fibrotic markers and studying of the pro-fibrotic signaling within lung PCTS, following TGF-β activation [54,55]. This study combined metabolomics, proteomics and SHG light microscopy imaging, which allowed the monitoring of fibrillar collagen deposition in a lung PCTS over a period of time without the need for fluorescent staining [54,55]. Such an approach could permit the high-throughput analysis of human PCTS, allowing for serial testing of multiple therapeutic regimens in single PCTS over time [54]. Thus, SHG imaging opens the avenue for efficient monitoring of collagen deposition, a hallmark of fibrosis and a major contributor to the creation of a hostile milieu in cancer that blocks immune cell infiltration, in PCTS. A combined approach of multiplex immunofluorescence and digital spatial profiling (DSP) has also been used recently in NSCLC PCTS, showcasing the complementary potential of such tools in accurately assessing metabolic and functional responses therapeutic agents in PCTS [57].

The biggest limitation of PCTS is the heterogeneity between slices of the same sample, which reflects tumor heterogeneity. To date, there is no standardized approach to cultivating tumor PCTS and studies are limited by their minimal biochemical characterization of the impact of slice preparation and cultivation conditions. It is difficult to systematically compare different slicing techniques and cultivation methods due to the inherent heterogeneity between tumor histology, patients and individual slices. A recent study of 108 resected primary or metastatic liver tumors revealed significant variability in growth and proliferative activity among different tumors, and significant metabolic heterogeneity at baseline between slices cut from the same tumor [16]. Adjacent slices demonstrated less heterogeneity compared to slices further apart. The authors demonstrated that some of these differences could be attributed to the amount of tumor in each slice as well as variation in metabolic activity between cells in each slice. This study demonstrates that, although some aspects of PCTS generation can be standardized to minimize baseline heterogeneity, other aspects, such as cell numbers and metabolic activity, cannot routinely be homogenized. Therefore, it may be necessary to normalize values based on cell numbers or activity, if possible. Additionally, given that adjacent slices are less heterogenous than slices taken from different areas of the tumor, experiments designed to study the effect of treatments may require an adjacent control slice next to every treatment slice.

Aside from inter- and intra-tumoral heterogeneity at baseline, studies show that the process of slicing and cultivating PCTS may induce changes in the tissue that render it biologically different from the original tumor. A study to comprehensively characterize the transcriptional changes in PCTS during cutting and culturing using RNA sequencing was recently published [58]. By comparing PCTS immediately after slicing with those in culture for 48 h, transcriptional changes induced by the slicing and culture process were measured. The findings confirmed that the preparation of PCTS causes significant injury to the PCTS, both by the mechanical trauma sustained during slicing as well as ischemia sustained prior to slicing. Additionally, various wound healing and fibrosis mechanisms in response to injury were similarly upregulated by slicing [58]. By comparing transcriptomic analysis of PCTS derived from healthy and diseased tissues from human liver, kidney and ileum at 0, 24 and 48 h, thousands of differentially (in the number and directionality) expressed genes were identified; however, there appeared to be some similarities between tissues. Transcripts encoding inflammatory cytokine IL-11 and extracellular matrix-degrading enzymes (MMP1, MMP3, MMP10) commonly had the highest fold upregulation in slices over time [58]. The most commonly downregulated genes were those encoding enzymes (PCK1, NAT8, FMP, GLYAT, HAO2), transporters (SLC13A1, SLC5A12, SLC34A1) and molecules involved in the immune response (CXCR1, FCGR2B, ACKR1) [57]. Interestingly though, despite some similarities, the effects of culture were organ and pathology specific. For example, certain genes were only upregulated in tissues derived from the ileum [58]. Additionally, when compared to diseased tissue PCTS, healthy tissue PCTS developed a stronger inflammatory response in culture [58].

A similar study looking at the effect of culture on gene transcription was performed in pancreatic ductal adenocarcinoma [59], where baseline FFPE tissue from five tumors and their matched cultured PCTS at 24, 48 and 72 h were compared. Interestingly, they found only a median of 12, 10 and 15 genes were upregulated and 15, 12 and 25 genes were downregulated at 24, 48 and 72 h compared with baseline FFPE samples [59].

These two studies highlight the transcriptional changes that occur during the slicing and culturing of PCTS. The changes appear to be both disease and organ specific, with different organs demonstrating different susceptibility to changes over time. Interestingly, even PCTS generated from the same organ, but of different patients, show variability in genomic changes during culture. The greater the number of changes, the less comparable PCTS are to their tissue of origin. The supplementation of culture media and manipulation of culture conditions have been proposed as mechanisms to overcome some of these changes [60,61,62], although the addition of supplements may in turn induce transcriptional changes in other genes in the PCTS. These studies also show that inter- and intra-tumoral heterogeneity remains a major limitation to the reproducibility of studies using PCTS. In view of this heterogeneity, a standardized approach to slicing and culturing PCTS from different organs and across disease types may not be feasible and studies using PCTS may require the optimization of parameters for cutting and culturing each specific organ and disease. Table 2 summarizes the main advantages and limitations of PCTS.

## 6. Conclusions

By retaining the landscape of the original tumor, PCTS are a physiologically relevant and important culture system for the study of solid tumor immunobiology. Data to date have also shown that this approach can be a relevant model across several tumor types for the individualized testing of drug susceptibility to improve clinical response rates. Having identified several limitations in the use of PCTS, recent and ongoing work from our team and others aims to better understand the potential of this platform as well as to optimize culture condition protocols to fully utilize it. Nevertheless, we anticipate that further work will eventually make PCTS useful for personalized clinical immunotherapy, especially in the case of adoptive cell therapy.

## Figures and Tables

**Table 1:** Summary of different microtomes available for tissue slicing.

Microtome Type	Tissues Optimally Sliced with Specific Microtome	Tissue Thickness	Advantages
Saw microtome	Hard specimens, such as teeth and bones	30 μm or higher	
Sledge microtome	Embedded samples	1–60 μm	
Rotary microtome	Thin, embedded samples (manual control)	0.5–60 µm	
Laser microtome	All types of samples	1 μm or higher	
Cryomicrotome	Frozen samples	2–50 μm	
Ultramicrotome	Extremely thin tissue slices	20–150 nm	Use with specialty microtomes
Krumdieck microtome (type of rotary microtome)	PCTS	100–500 μm	First microtome to be routinely used for PCTS generation. Best for glioblastoma PCTS
Vibrating microtome(Vibratome)	Fixed and PCTS	Fixed: >10 μm PCTS: 30–1000 μm	- Less pressure and stress to the PCTS than with the Krumdieck microtome - Precise and reproducible. - Better preservation of tissue integrity
Compresstome	Fixed and PCTS	Fixed: >10 μm PCTS: 30–1000 μm	- Milder “compression” of tissue, reduced tissue damage and shearing - No uneven slicing or vibration-related artefacts - 5 times faster in tissue slicing than the Vibratome

**Table 2:** Main advantages and limitations of PCTS.

Advantages	Limitations
Retain the 3D architecture of the original tumor, including stromal and immune cell compartments.	Lack of vascularization does not permit long-term culture
Quick, easy, and relatively inexpensive to generate and culture	Cannot currently be frozen or biobanked
Allow studies on the immunobiology of tumors	Any experiments need to fit within the short-term culture timeframe of ~one week
Drug screening of immunotherapeutic agents is possible	Low throughput platform with no direct multi-omics possible
Permit several assay applications following culture (IHC, flow cytometry, confocal, microscopy, sequencing and supernatant readouts)	Inter- and intra-tumoral heterogeneity observed
	Organ/tumor-specific transcriptional changes observed following slicing

## Data Availability

Not applicable.
